# Multi-Analytical Approach Reveals Potential Microbial Indicators in Soil for Sugarcane Model Systems

**DOI:** 10.1371/journal.pone.0129765

**Published:** 2015-06-09

**Authors:** Acacio Aparecido Navarrete, Tatiana Rosa Diniz, Lucas Palma Perez Braga, Genivaldo Gueiros Zacarias Silva, Julio Cezar Franchini, Raffaella Rossetto, Robert Alan Edwards, Siu Mui Tsai

**Affiliations:** 1 Cell and Molecular Biology Laboratory, Center for Nuclear Energy in Agriculture CENA, University of São Paulo USP, Piracicaba, SP, Brazil; 2 Computational Science Research Center, San Diego State University, San Diego, California, United States of America; 3 Brazilian Agricultural Research Corporation, Londrina, PR, Brazil; 4 São Paulo's Agency for Agribusiness Technology, Piracicaba, SP, Brazil; 5 Department of Computer Science, San Diego State University, San Diego, California, United States of America; 6 Division of Mathematics and Computer Science, Argonne National Laboratory, Argonne, Illinois, United States of America; USDA-ARS, UNITED STATES

## Abstract

This study focused on the effects of organic and inorganic amendments and straw retention on the microbial biomass (MB) and taxonomic groups of bacteria in sugarcane-cultivated soils in a greenhouse mesocosm experiment monitored for gas emissions and chemical factors. The experiment consisted of combinations of synthetic nitrogen (N), vinasse (V; a liquid waste from ethanol production), and sugarcane-straw blankets. Increases in CO_2_-C and N_2_O-N emissions were identified shortly after the addition of both N and V to the soils, thus increasing MB nitrogen (MB-N) and decreasing MB carbon (MB-C) in the N+V-amended soils and altering soil chemical factors that were correlated with the MB. Across 57 soil metagenomic datasets, *Actinobacteria* (31.5%), *Planctomycetes* (12.3%), *Deltaproteobacteria* (12.3%), *Alphaproteobacteria* (12.0%) and *Betaproteobacteria* (11.1%) were the most dominant bacterial groups during the experiment. Differences in relative abundance of metagenomic sequences were mainly revealed for *Acidobacteria*, *Actinobacteria*, *Gammaproteobacteria* and *Verrucomicrobia* with regard to N+V fertilization and straw retention. Differential abundances in bacterial groups were confirmed using 16S rRNA gene-targeted phylum-specific primers for real-time PCR analysis in all soil samples, whose results were in accordance with sequence data, except for *Gammaproteobacteria*. *Actinobacteria* were more responsive to straw retention with *Rubrobacterales*, *Bifidobacteriales* and *Actinomycetales* related to the chemical factors of N+V-amended soils. *Acidobacteria* subgroup 7 and *Opitutae*, a verrucomicrobial class, were related to the chemical factors of soils without straw retention as a surface blanket. Taken together, the results showed that MB-C and MB-N responded to changes in soil chemical factors and CO_2_-C and N_2_O-N emissions, especially for N+V-amended soils. The results also indicated that several taxonomic groups of bacteria, such as *Acidobacteria*, *Actinobacteria* and *Verrucomicrobia*, and their subgroups acted as early-warning indicators of N+V amendments and straw retention in sugarcane-cultivated soils, which can alter the soil chemical factors.

## Introduction

Quantitative and qualitative changes in soil characteristics are expected when using different types of soil agricultural management, which leads to different nutrient availability to the soil that will determine, favor or inhibit the establishment of different microbial groups [1–3]. Organic and inorganic fertilizer amendments are primarily used to increase nutrient availability to plants, but they can also affect soil microbial community composition [4,5].

Soil management practices used in sugarcane agriculture in Brazil, which is the largest world’s producer of sugarcane, require synthetic mineral fertilizers (nitrogen/phosphorus/potassium—NPK) [6] and full recycling of waste products from ethanol production to sugarcane fields in the form of organic fertilizer [7]. Vinasse is a by-product of the sugar-ethanol industry produced in large quantities, and it is composed of water, organic matter, and mineral elements [8]. Since the 1960’s, vinasse (V) has been used as a liquid fertilizer in the sugarcane fields of Brazil to solve the ecological problem of its disposal within the environment. Studies from the late 1980’s have recommended the use of N fertilizer in combination with V [9] in sugarcane fields, and a more recent study has recommended the use of N fertilizer with straw retention [10]. Although N fertilization use combined with V and straw retention improves soil fertility and sugarcane productivity, there is a lack of information on the impacts of such combinations on the microbiological properties of tropical soils.

Recent studies have demonstrated metagenomic, phylogenetic and physiological responses of microbial communities across N gradients in soil [11–13]. However, the effects on the soil microbial community composition of N fertilizer alone or in combination with waste products from sugar-ethanol production used as organic fertilizer have not been reported for sugarcane agriculture in Brazil. Advances in next-generation DNA sequencing methods in combination with traditional microbiological and chemical analyses of soil factors may be used to define a biologically relevant assay to estimate the potential effects of fertilizer use and straw retention on indigenous microbial communities.

Organic and inorganic amendments and straw retention used as a surface ‘blanket’ are likely to affect the biological and chemical characteristics of sugarcane soils; therefore, we investigated the soil microbial community along with processes occurring in the soil under the sugarcane production systems commonly used in southeast Brazil. First, we hypothesized that changes in soil microbial biomass (MB) carbon (MB-C) and nitrogen (MB-N) may be correlated with fertilizer-induced CO_2_-C and N_2_O-N emissions from sugarcane-cultivated soils under organic and inorganic amendments and straw retention as well as soil chemical factors arising in these agricultural soils. Second, based on a more detailed taxonomic analysis of the soil microbial community using high-throughput DNA sequencing, we hypothesized that taxonomic groups of bacteria can respond to incorporation of N and V as fertilizer into the sugarcane-cultivated soils and sugarcane straw-blanket effects in these soils. For these purposes, we used a multi-analytical approach in sugarcane-cultivated soils in a short-term greenhouse experiment that incorporated measurements of carbon dioxide (CO_2_) and nitrous oxide (N_2_O) emissions from soil, a chemical factor analysis of the soil samples, and a survey of the soil microbial community using methods to determine the MB and abundance of taxonomic groups of bacteria (fumigation-extraction procedure, shotgun metagenomic sequencing and real-time quantitative PCR). The results of this study are particularly important for the evaluation of management practices related to fertilizer use in sugarcane-cultivated soils.

## Materials and Methods

### 2.1. Experimental design, treatments and soil sampling

The sugarcane (*Saccharum* spp.) variety CTC-02 is characterized by medium-late maturation, high productivity and longevity, and it was grown from April until December 2013 (250 days) in a greenhouse mesocosm experiment. The influence of environmental parameters, such as moisture regime, soil type and fertilizer management, were normalized on the growth conditions for *in vitro* plants obtained via tissue culture techniques. Podzolic dark red soil (clay loam texture) was collected from the 0 to 20 cm topsoil layer in the experimental field of the Areão Farm at ESALQ/USP, Piracicaba, São Paulo, Brazil (22° 42' 30" S e 47° 38' 00" W). Eighteen mesocosms in plastic pots (100 L) were filled with 90 kg of soil, which was placed over a 15 cm layer of washed stones. Mineral fertilization that is common in all mesocosms and consisting of 150 kg ha^-1^ P_2_O_5_ (triple superphosphate) and 80 kg ha^-1^ KCl (potassium chloride) was used in this experiment. Six treatments and three replications were used in a completely randomized design. Mineral fertilizer was applied in the form of urea (450 g N kg^-1^) to the 0–10 cm topsoil layer at a rate of 60 kg N ha^-1^ in treatments containing N fertilizer. A small shovel was used to mix the urea to the soil avoiding losses by volatilization. Vinasse is a liquid residue of ethanol distillation, and it was applied to the soil at a rate of 0.06 L kg^-1^ (120 m^3^ ha^-1^) as a source of K in addition to organic matter and other nutrients. An equivalent water volume was applied in treatments without V. The experiment consisted of two conditions of soil-surface straw blanket as follows: surface blanket with sugarcane straw (10 t ha^-1^) and uncovered surface. The straw blanket consisted of dry and chopped leaves from adult sugarcane plants. The KCl dosage was calculated minus the equivalent input of K in case of straw blanket and V treatments according to previous measurements of K content in sugarcane straw and V samples. Accordingly, the experiment included the following treatments: N, nitrogen fertilizer; N+S, N fertilizer and straw blanket; N+V, N and vinasse as fertilizers; N+V+S, N and V as fertilizers and straw blanket; C, excluding any N, V fertilizer and straw blanket (control); and C+S, excluding any N and V fertilizer and including straw blanket. In order to provide nutrients for the growth of the sugarcane plants until ripening phase, three applications of fertilizers were defined based on plant deficiency symptoms and fertilizer-induced CO_2_-C and N_2_O-N emissions from the soil. The soil moisture was monitored daily in each mesocosm by using soil moisture sensor (Extech MO750, Nashua, NH, USA) in order to maintain the humidity at the 20%.

Ten sugarcane plants were grown in each mesocosm, and only two sugarcane plants were left in each mesocosm until the end of the experiment. Sugarcane plants were removed in pairs from each mesocosm at 50, 90, 150 and 210 days after the first soil fertilization to maintain the root system under the limit capacity of the mesocosm.

For each mesocosm, soil samples were collected before the first fertilization and on the maximum and minimum gas flux time points over time in each of three applications of fertilizer for chemical factor analysis in addition to MB-C and MB-N determinations. Soil samples for DNA isolation were collected before the first fertilization and during the maximum CO_2_-C and N_2_O-N emissions from soil in each of the three applications of fertilizer. All of the soil samples were collected from the 0 to 10 cm topsoil layer using a cylindrical sampler (2 cm diameter) after removing the straw blanket when present. Soil samples for chemical analysis were immediately processed after sampling. Soil samples were stored at 4°C for MB analyses, which were performed within 2 weeks after sampling. Soil samples for DNA isolation were transported to the laboratory under ice and stored at -20°C until processing within 72 h after sampling.

### 2.2. Sample collection and calculation of CO_2_-C and N_2_O-N emissions from soil

Gas samples were collected over time after each application of fertilizer to the soil. Samples were collected using chambers (20 cm diameter, 20 cm height, and 0.0067 m^3^) installed at the center of the surface area in each mesocosm by inserting the base into the soil to a depth of 3 cm. The chambers used for gas sampling consisted of an aluminum pipe that served as a base, PVC cap that fit snugly on the base and small valve to prevent overheating and subsequent increases in the chamber’s internal pressure. During each sampling event, four samples were collected from each chamber for a period of 60 min. The first sample was collected 1 min after the chamber was closed, and the remaining samples were collected after 20, 40, and 60 min. Gas samples were collected using a 60 ml BD plastic syringe (Becton, Dickinson and Co., Franklin Lakes, NJ, USA), and the samples were immediately placed in 30 ml previously evacuated glass vials closed with rubber stoppers (Bellco Glass, Vineland, NJ, USA). Samples were analyzed by gas chromatography within five days of collection. Overall, 2,664 samples were collected in 18 chambers during 37 sampling events. The first sampling occurred immediately after the first fertilizer application and on the day after, whereas the following samplings were spaced first by 2 days and then by 3 days until the 150th day after fertilization. After the second and third fertilizer applications to the soil, sampling became less frequent, occurring immediately after the fertilizer applications and spaced by 2 days until the 7th day after fertilization and then approximately once every 2 weeks. Determination of CO_2_ and N_2_O, using nitrogen as gas flow, was performed by gas chromatography (SRI 8610C Model, Torrance, CA, USA) with flame ionization detector (FID) and electron capture detector (ECD), respectively, and HayeSep-D and-N packed columns at 81°C and 20 ml/min. In FID, the samples were undergone a combustion in a hydrogen (5.0)/synthetic air flame. Prior to detection, CO_2_ was reduced to CH_4_ using a methanizer. Gas concentrations were calculated by comparing peak areas of the samples to those of commercially prepared standards (White Martins, Piracicaba, Brazil). Fluxes were calculated by a linear fit of concentration data as a function of the incubation time [14]. The CO_2_-C and N_2_0-N emission rates for each sampling event were computed using a linear regression based on the curve generated from the gas values measured along the 60 min intervals.

### 2.3. Analysis of soil chemical factors

Soil samples were air dried and sieved through a 0.149 mm for total C and N determination by dry combustion on a LECO CN elemental analyzer at the Center for Nuclear Energy in Agriculture, University of São Paulo, Brazil. The fertility status of the soil from each soil sample was assessed as described in Navarrete et al. [2], with organic matter (OM) determined according to Camargo *et al*. [15] at the Soil Fertility Laboratory, Department of Soil Sciences, University of São Paulo. The evaluated soil fertility factors included pH, potential acidity (H + Al), Ca, Mg, P, K, S, available micronutrients (Fe, Mn, Zn and Cu), exchangeable bases (EB; the sum of Ca, Mg and K), cation exchange capacity (CEC), and base saturation (V).

### 2.4. Soil microbial biomass

The contents of soil MB-C and MB-N were evaluated by the fumigation-extraction method using Kc values of 0.33 and 0.54, respectively [16, 17]. The carbon content in the extracts was determined using a spectrophotometer according to the method of Bartlett and Ross [18]. Nitrogen content in the same fractions was evaluated by the Kjeldahl method followed by the spectrophotometric determination of NH_4_–N using the indophenol blue method [19].

### 2.5. Isolation of DNA from soil and high-throughput sequencing of soil metagenome

DNA was extracted from 250 mg (wet weight) of 57 soil samples (3 samples taken before the first fertilization + 3 samples x 6 experimental treatments x 3 applications of fertilizer) using the Power Lyzer Power Soil DNA Isolation Kit (Mo Bio Laboratories Inc., Carlsbad, CA, USA) according to the manufacturer’s instructions. The DNA extracts were stored at −20°C until use.

Soil DNA samples were used to prepare libraries using the MiSeq Reagent Kit v.2 (500 cycles; Illumina, San Diego, CA, USA) for shotgun metagenomic sequencing in a MiSeq Personal Sequencing System (Illumina, San Diego, CA, USA). In summary, we sequenced a subset of the original 57 samples and captured an average of 105.5 MB of genomic sequences per sample (S1 Table).

### 2.6. Data preprocessing and taxonomic annotation of sequences from soil metagenomic datasets

First, paired-end reads were merged using FLASH version 1.2.5 [20] to produce consensus sequences and increase the annotation accuracy. Second, low-quality bases (quality score lower than 20) from merged and unmerged sequences were trimmed from both ends using the Phred algorithm with SeqClean script (http://www.bioinformatics.org/). Merged and unmerged trimmed sequences were concatenated into a single file for each metagenomic dataset, which are available through the Metagenomics Rapid Annotation (MG-RAST) server (http://www.metagenomics.anl.gov) under project accession ‘Metagenomes of sugarcane soils–CENA USP’ and accession numbers 4582104.3 to 4582153.3.

A taxonomic analysis of the unassembled DNA sequences was performed with FOCUS [21], a fast composition-based method, using the database of only bacterial genomes. Initially, a table of the relative abundance of hits was generated for each individual taxon for each dataset at the phylum level. An order level was then analyzed for *Actinobacteria* using the same database. In addition, all 57 metagenomic datasets were aligned by BLASTN 2.2.28+ [22] using an e-value threshold of ≤10^−5^ against a database of 16S rRNA gene sequences of *Acidobacteria* and *Verrucomicrobia* (14,695 sequences of *Acidobacteria* and 24495 sequences of *Verrucomicrobia*) downloaded from the RDP database (http://rdp.cme.msu.edu/). The class level was used to analyze *Acidobacteria* and *Verrucomicrobia*. Only the best hit for each query sequence was used in the count.

### 2.7. Statistical analysis of metagenomic datasets

A Tukey’s test was used to determine the significance of the differences in relative abundance of taxonomic groups of bacteria between soil samples from mesocosms with and without straw blankets within each application of fertilizer to the soil. The statistical comparison of soil samples was performed using Statistica v. 10.0 software (Statsoft Inc., Tulsa, OK, USA). A repeated measures analysis of variance (rANOVA) was performed using the GLM procedure from Statistical Analysis System v. 9.3 (SAS, Cary, NC, USA) to assess the effects of factors such as time (repeated applications of fertilizer) and experimental treatments on the relative abundance of taxonomic groups of bacteria along with their interactions. Heat maps were generated, using a homemade python script (S1 File) and the matplotlib plotting library [23], from the predicted relative abundance of groups of bacteria as computed by FOCUS software in order to assess straw blanket effect on group-specific bacterial communities. The heat maps used the Euclidean distance as distance method. The explicit relationship between the relative abundance of group-specific bacterial taxonomical classes or orders and soil chemical factors was examined by constrained ordination generated by a redundancy analysis (RDA) performed using CANOCO 4.5 [24].

### 2.8. Quantitative real-time PCR assays for group-specific bacterial communities

Quantitative real-time PCR (qPCR) using the 16S rRNA gene as a biomarker was performed to assess the abundance of acidobacterial, actinobacterial, γ-proteobacterial and verrucomicrobial communities in the same 57 soil samples used for shotgun metagenomic sequencing. Amplicons of *Acidobacteria capsulatum* (DSMZ 11244), *Gordonia* spp. (DSM 11192), *Xanthomonas campestris* (DSMZ 3586) and *Verrucomicrobia spinosum* (DSMZ 4136) were used as standards. DNA standard curves were generated by dilution series of 10^3^ to 10^8^ copies μl^-1^ using duplicate 10-fold dilutions of *A*. *capsulatum*, *Gordonia* spp., *X*. *campestris* and *V*. *spinosum* standard DNA. The following primer pairs were used for qPCR of 16S rRNA gene fragments from *Acidobacteria*, *Actinobacteria*, γ-*Proteobacteria* and *Verrucomicrobia*: Acid31 (5’-GATCCTGGCTCAGAATC-3’) [25]/Eub518 (5’-ATTACCGCGGCTGCTGG-3’) [26] for *Acidobacteria*; Act920F3 (5’-TACGGCCGCAAGGCTA-3’) [27]/Act1200R (5’-TCRTCCCCACCTTCCTCCG-3’) [27] for *Actinobacteria*; 1080F (5’-TCGTCAGCTCGTGAAATT-3’) [27]/1202R (5’-CGTAAGGGCCATGATG-3’) [27] for γ-*Proteobacteria*; and Ver53 (5’-TGGCGGCGTGGWTAAGA-3’) [28] and Eub518 for *Verrucomicrobia*. Each 25 μl reaction contained 12.5 μl of absolute qPCR SYBR green 2x reaction mix (Abgene, Epsom, UK), 1.25 μl of each primer (30 μM), 2.5 μl of bovine serum albumin (BSA; 10 mg ml^-1^) and 50 ng of template DNA. PCR conditions for *Acidobacteria* and *Verrucomicrobia* were performed as described by Fierer et al. [29] with the following modifications: annealing temperatures of 49°C for *Acidobacteria* and 60°C for *Verrucomicrobia*; and forward primer (Ver53) in the case of *Verrucomicrobia*. PCR conditions for *Actinobacteria* and γ-*Proteobacteria* were as described by De Gregoris et al. [27]. PCR amplifications and product quantification were performed using the StepOnePlus^TM^ Real Time PCR System (Applied Biosystems, Foster, CA, USA). A melting curve analysis of amplicons was performed to confirm that the fluorescence signals originated from specific amplicons and not from primer-dimers or other artifacts. Automated analyses of PCR amplicon quality (for example, PCR baseline subtraction and cycle threshold (Ct) setting to the linear amplification phase) and quantity were performed with StepOnePlus^TM^ Real Time software v.2.2 (Applied Biosystems, Foster, CA, USA). Statistical analyses of qPCR data were performed using the Statistica v.10.0 software (StatSoft Inc., Tulsa, OK, USA). A Tukey’s test was used to determine the significance of the differences between soil samples from mesocosms with and without straw blankets for each group-specific bacterial community.

## Results

### 3.1. Soil microbial biomass correlations with gas emissions and soil chemical factors

**Fig 1:**
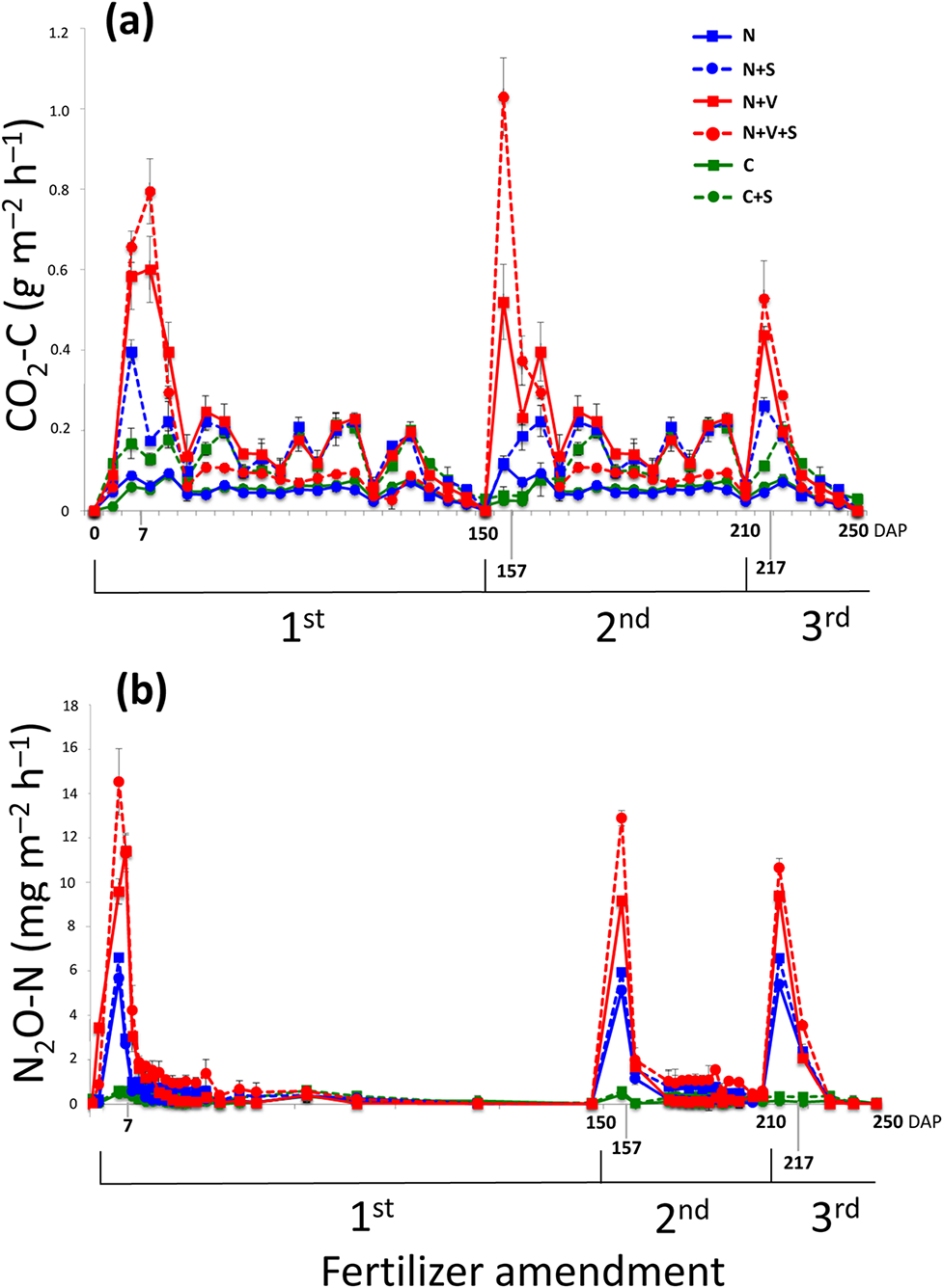
CO_2_-C (a) and N_2_O-N (b) emission rates from soil over time in each of three applications of fertilizer. The different treatments are represented as follows: N, nitrogen fertilizer; N+S, N fertilizer and straw blanket; N+V, N and vinasse as fertilizer; N+V+S, N and V as fertilizer and straw blanket; C, excluding any N, V and straw blanket (control); C+S, excluding any N and V fertilizer and including straw blanket. The graph represents the average flux based on gas samples collected from three different mesocosms for each treatment during each sampling event. The standard deviation is shown in the graph. Axis X shows the time based on days after planting (DAP).

The CO_2_-C and N_2_O-N emission rates from soil varied among the experimental treatments and defined the maximum and minimum gas flux time points for each application of fertilizer (Fig 1). In general, CO_2_-C and N_2_O-N emission rates increased until the seventh day after each fertilization application and subsequently declined regardless of treatment in each of the three applications of fertilizer. The minimum emission rates occurred at 150, 60 and 40 days after fertilization for the first, second and third application of fertilizer, respectively.

Increases in CO_2_-C and N_2_O-N emissions were identified immediately after the addition of N and V to the soils, with concomitant increase in MB-N and decrease in MB-C (Table 1; S2 Table). Straw retention showed the highest gas emissions from the soil, especially in both the N- and N+V-amended soils (Fig 1); however, soil MB did not show differences between soils with and without a straw blankets (S2 Table).

**Table 1:** Spearman’s rank correlation between microbial biomass carbon and nitrogen and gas emissions and chemical factors of cultivated-sugarcane soils.

Treatments	CO_2_-C	N_2_O-N	Ctot	Ntot	OM	Sulfur	Potassium	pH
	Carbon-Microbial biomass							
N								
N+S								
N+V	-0.856*	-0.872*		-0.941**	-0.932**	-0.985***	-0.942*	0.882*
N+V+S	-0.865*	-0.889*		-0.882*	-0.811*	-0.997**	-0.991*	0.876*
C									
C+S			-0.885*						
	Nitrogen-Microbial biomass							
N								
N+S					0.840*			
N+V	0.807*	0.841*		0.985*	0.998**	-0.973*	-0.909*	-0.794*
N+V+S	0.811*	0.067*		0.794*	0.986**	-0.988*	-0.912*	-0.971**
C				0.957***	0.942*			
C+S			-0.857*	0.985***	0.996**			

N = nitrogen as fertilizer; V = *vinasse* as fertilizer; S = straw blanket; C = control—without any N and V fertilizer; Ctot = total soil carbon; Ntot = total soil nitrogen; OM = organic matter

CO_2_-C and N_2_O-N emission rates shown in Fig 1

Soil chemical results shown in S1 and S2 Tables

Significant levels for the Spearman’s rank coefficients are indicated at the

*P < 0.05

**P < 0.005

***P < 0.0005 level

With regard to the correlation between MB and the chemical factors of the sugarcane-cultivated soils, MB-C and MB-N were negatively correlated with total C in the control soils with a straw blanket (Table 1). In addition, MB-C was negatively correlated with total N and organic matter in the N+V-amended soils (Table 1). Although MB-C increased after the first N and V applications to the soil, decreased MB-C was found after the second application of these fertilizers to the soil (S2 Table). Over time for all treatments, MB-C and MB-N trended to decrease, whereas total soil C trended to increase (S2 Table). Positive correlations were observed between MB-N and total N and organic matter for N+V-amended and control soils (Table 1).

Correlations between MB and other soil chemical factors linked to soil fertility in sugarcane-cultivated soils were found with the sulfur and K contents (Table 1). These correlations were negative for both MB-C and MB-N, and they were present only for N+V-amended soils. MB-C and MB-N were positively and negatively correlated with soil pH in the N+V-amended soils, respectively (Table 1). The soil pH increased over time in the N+V-amended soils for both surface blanket conditions (S3 Table). MB was not significantly correlated with the exchangeable bases in soil.

### 3.2. Responses of taxonomic groups of bacteria to soil amendments

**Fig 2:**
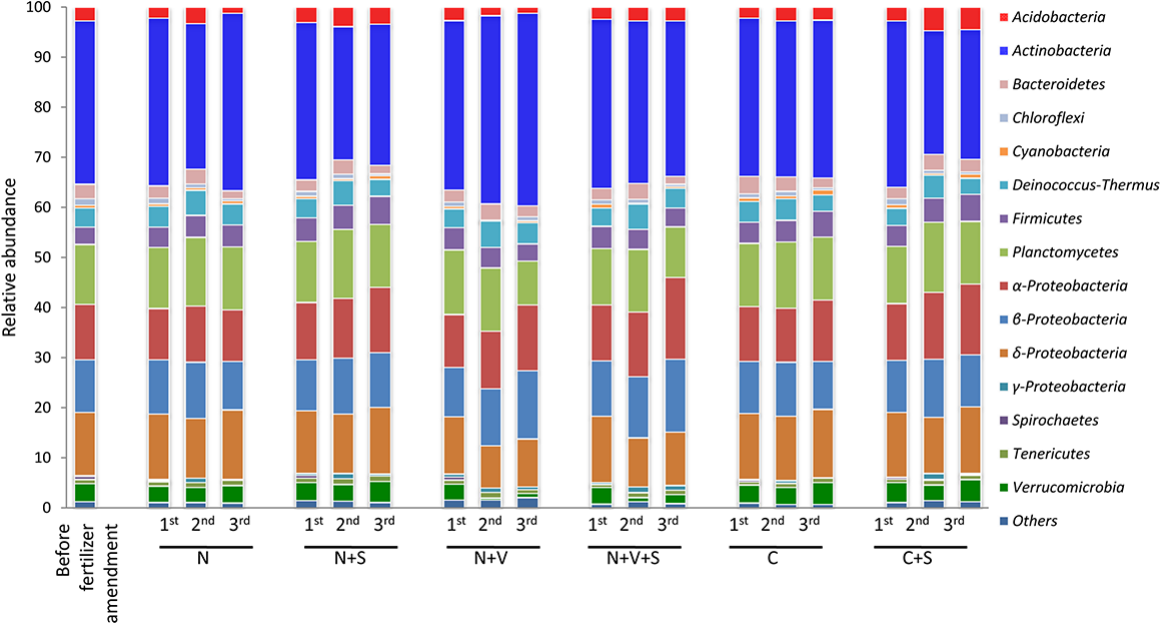
The 100% stacked column chart of the relative abundances of bacterial groups from metagenomic sequencing data in each of three applications of fertilizer. The different treatments are represented as follows: N, nitrogen fertilizer; N+S, N fertilizer and straw blanket; N+V, N and vinasse as fertilizer; N+V+S, N and V as fertilizer and straw blanket; C, excluding any N, V and straw blanket (control); C+S, excluding any N and V fertilizer and including straw blanket. The value of each bacterial group percentage is the mean of soil samples collected from three different mesocosms (S4 Table).

Shotgun sequencing of soil DNA from the 57 soil samples (DNA samples described in subsection 2.5) resulted in approximately 13.5 million merged sequence reads and 8.7 million non-merged sequence reads after the quality-based filtering procedure (S1 Table). Sequence data were examined in soils to estimate the relative abundance of bacteria in taxonomic groups (Fig 2). The most dominant bacterial groups in all soil samples over time in the experiment were *Actinobacteria*, *Planctomycetes*, *Alphaproteobacteria*, *Betaproteobacteria* and *Deltaproteobacteria*. There were significant differences (*p*<0.05) in the relative abundance of taxonomic groups of bacteria between soils that were uncovered and covered with a straw blanket (S4 Table). These differences were found in the N-amended, N+V-amended, and control soils, and they became more evident in the N-amended and N+V-amended soils to the extent that were made N and V applications to the soil. In general, the straw blanket altered the soil bacterial community composition by increasing the relative abundance of *Acidobacteria*, γ-*Proteobacteria* and *Verrucomicrobia* and by decreasing the relative abundance of *Actinobacteria* (S4 Table). *Acidobacteria*, *Actinobacteria* and *Verrucomicrobia* revealed the most significant effect from the repeated applications of fertilizer to the soil and experimental treatments according to the results from rANOVA (Table 2). Others bacterial groups such as α- β- δ- and γ-*Proteobacteria* also revealed statistical significance (*p* < 0.0001) for time or treatment, but not for time and treatment interactions based on rANOVA (Table 2).

**Table 2:** Repeated measures ANOVA (rANOVA) of the relative abundance of bacterial groups as a function of time (applications of fertilizer) and experimental treatments, along with interaction.

**Bacterial groups**	Time		Treatment		Time x Treatment	
	F	***p***	F	***p***	F	***p***
*Acidobacteria*	46.84	<.0001	18.32	<.0001	6.02	<.0001
*Actinobacteria*	54.43	<.0001	16.21	<.0001	6.07	<.0001
*Bacteroidetes*	7.42	0.003	3.16	0.047	3.63	0.005
*Chloroflexi*	7.48	0.003	0.82	0.556	1.37	0.250
*Cyanobacteria*	31.12	<.0001	7.63	0.002	4.58	0.001
*Deinococcus-Thermus*	27.43	<.0001	10.30	0.0005	1.00	0.470
*Firmicutes*	2.46	0.107	8.53	0.001	4.68	0.0009
*Planctomycetes*	21.07	<.0001	9.11	0.0009	4.02	0.002
*α-Proteobacteria*	20.81	<.0001	14.78	<.0001	2.56	0.029
*β-Proteobacteria*	7.77	0.002	8.07	<.0001	4.81	0.0002
*δ-Proteobacteria*	18.20	<.0001	11.06	0.0004	4.68	0.0002
*γ-Proteobacteria*	18.40	<.0001	4.45	0.016	2.25	0.050
*Spirochaetes*	26.56	<.0001	2.57	0.084	1.27	0.300
*Tenericutes*	20.75	<.0001	2.00	0.150	1.89	0.098
*Verrucomicrobia*	40.70	<.0001	21.50	<.0001	7.80	<.0001
Others	9.45	0.0009	2.44	0.095	0.88	0.561

Degrees of freedom (DF): Time: DF = 2; Treatment: DF = 5; and Time x Treatment: DF = 10

The Tukey’s test performed on the qPCR data targeting 16S rRNA gene fragment abundances for *Acibobacteria*, *Actinobacteria*, γ-*Proteobacteria* and *Verrucomicrobia* showed the same trends as were revealed by sequencing for these taxonomic groups of bacteria, except for γ-*Proteobacteria* (Table 3). The 16S rRNA gene fragment abundances for *Acidobacteria* and *Verrucomicrobia* were higher in soils with a straw blanket compared with uncovered soils. The opposite result was found for *Actinobacteria* based on the 16S rRNA gene fragment abundance in the same soils.

**Table 3:** Absolute abundance of group-specific bacterial community measured by quantitative real-time PCR before fertilizing and on the maximum CO_2_-C and N_2_O-N emissions from soil over time in three applications of fertilizer.

Bacterial groups	Before fertilizer amendment	First fertilizer amendment (at Sowing– 0 DAP)				Control	
		N	N+S	N+V	N+V+S	C	C+S
*Acidobacteria*	19.3±1.4	18.2* a^‡^A^§^±1.6^†^	20.8 aA±1.8	24.3 aA±7.1	17.9 aA±0.6	18.2 aA±1.6	20.8 aA±1.8
*Actinobacteria*	36.6±5.7	38.9 aA±4.8	36.5 aA±2.5	34.9 aA±7.8	37.9 aA±9.3	34.5 aA±8.4	30.0 aA±8.9
γ-*Proteobacteria*	1.1±0.3	1.3 aA±0.3	1.4 aA±0.2	1.4 aA±0.4	1.2 aA±0.3	0.9 aA±0.4	0.9 aA±0.3
*Verrucomicrobia*	3.3±1.0	3.1 aA±1.4	3.2 aA±1.2	3.0 aA±1.8	3.1 aA±1.1	3.4 aA±1.7	3.5 aA±1.2
		**Second fertilizer amendment (150 DAP)**				**Control**	
*Acidobacteria*		17.1 aA±1.3	20.8 aA±3.8	10.4 aA±5.2	15.5 aB±3.7	18.6 aA±2.5	24.8 bA±3.4
*Actinobacteria*		39.6 aA±2.9	37.4 aA±2.2	37.7 aB±1.3	32.1 aB±3.4	31.3 aA±2.1	25.4 bA±2.1
γ-*Proteobacteria*		1.9 aA±0.4	2.0 aA±0.5	1.8 aA±0.3	2.1 aA±0.5	1.3 aA±0.3	1.5 aA±0.5
*Verrucomicrobia*		2.5 aA±0.2	2.3 aA±0.7	1.3 aB±0.2	1.4 aB±0.2	2.3 aA±0.7	2.3 aA±0.3
		**Third fertilizer amendment (210 DAP)**				**Control**	
*Acidobacteria*		12.9 aB±1.0	17.5 bA±3.1	11.3 aB±1.2	15.9 bA±2.0	18.2 aA±1.5	17.0 bA±1.0
*Actinobacteria*		43.7 bA±1.4	39.8 aB±1.1	49.1 bB±2.0	43.7 aB±2.4	34.2 aA±1.6	30.8 bA±1.3
γ-*Proteobacteria*		1.1 aA±0.3	1.3 aA±0.4	1.4 aA±0.3	1.5 aA±0.3	1.0 aA±0.2	1.1 aA±0.3
*Verrucomicrobia*		2.3 aA±0.7	3.9 bA±0.8	1.5 aB±0.4	2.6 bB±0.3	3.4 aA±0.7	4.1 aA±0.1

DAP = days after planting

N = nitrogen as fertilizer; V = *vinasse* as fertilizer; S = straw blanket; C = control—without any N and V fertilizer

The values are expressed as 10^7^ 16S rRNA gene copies per gram of soil

*Average for each of three replicates of soil

†Standard deviation of the average for each of three replicates of soil

Tukey’s test was performed separately for each of three fertilizer applications. Samples with and without straw blankets were contrasted for treatments equally fertilized (‡), and also for fertilized treatments and control soils under the same straw blanket condition (§)

Values with the same lower or upper-case letters were not significantly different (*p*<0.05) based on upon a Tukey’s test between contrasted samples

**Fig 3:**
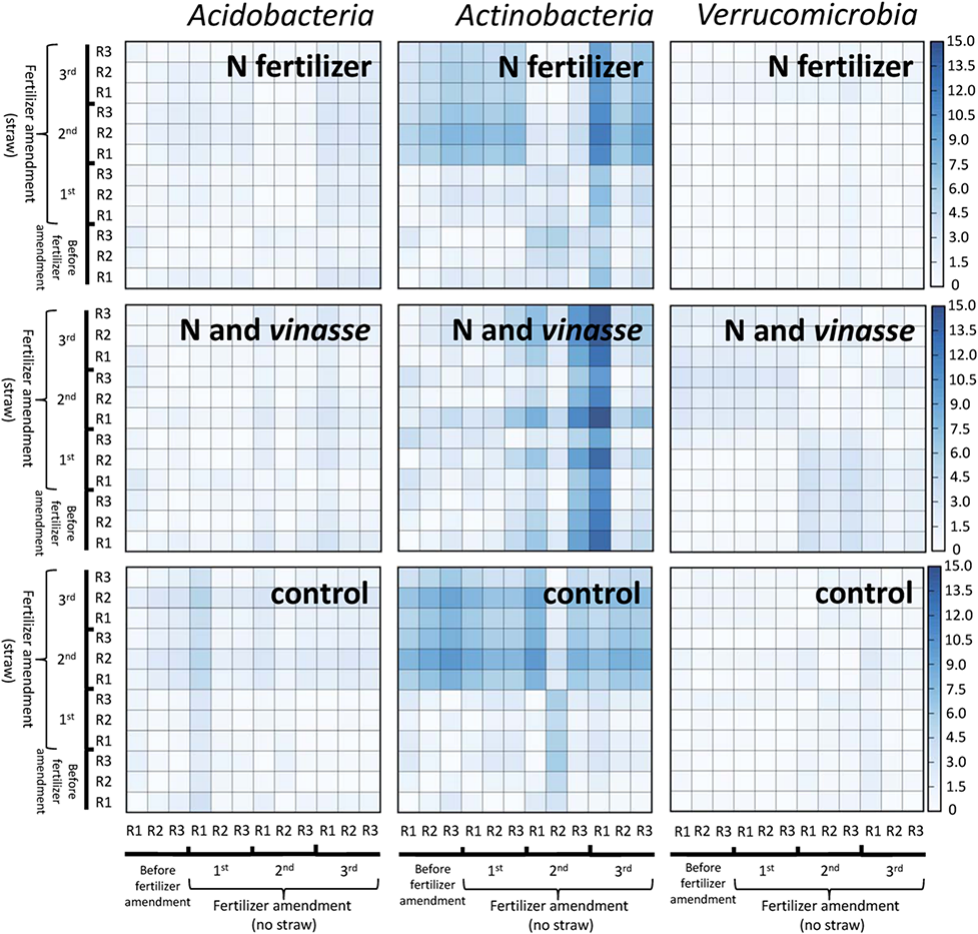
Taxonomic heat maps based on the Euclidean distance of acidobacterial, actinobacterial and verrucomicrobial communities as a percent of the total bacterial sequences as computed by FOCUS software. The distance matrix was obtained based on soil metagenomics datasets from samples with and without sugarcane straw blanket. Treatment excluding any N and V fertilizer is represented as the control. R represents the replication of metagenomic profiling of soil samples based on group-specific bacterial communities.

Heat maps were generated from sequencing data based on the relative abundance of bacteria into taxonomic groups that revealed consistent response based on sequence data, qPCR data and rANOVA results (i.e. *Acidobacteria*, *Actinobacteria* and *Verrucomicrobia*). Based on heat map graphical representation, actinobacterial community revealed a greater response to straw blankets compared with the response of acidobacterial and verrucomicrobial communities (Fig 3). The straw blanket effect on the actinobacterial community was shown for N-amended, N+V-amended, and control soils (Fig 3). The N+V-amended soils showed the highest Euclidean distance values (15.0) for the actinobacterial community after the third application of fertilizer to the soil compared with the straw blanket conditions. However, an early-warning response was shown for the actinobacterial community in N-amended and control soils regarding the straw blanket effects.

**Fig 4:**
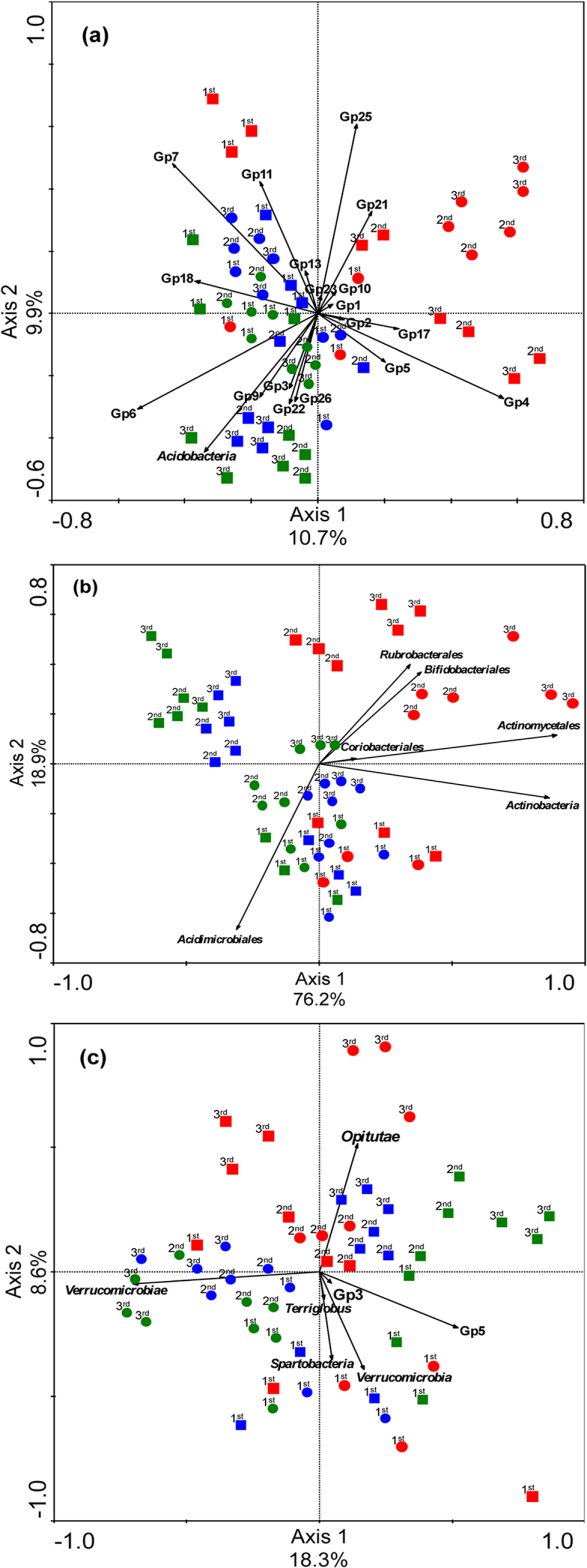
Constrained ordination diagram for sample plots in the first two redundancy analysis (RDA) axes. The axes were based on the soil chemical factors and their relationship with the relative abundance of *Acidobacteria* at the phylum and class (subgroup) levels (a), *Actinobacteria* at the phylum and order levels (b), and *Verrucomicrobia* at the phylum and class levels (c). Squares represent soils with a straw blanket, and circles represent uncovered soils. N-amended soils are represented in blue. N+V-amended soils are represented in red. Soils excluding any N and V fertilizer are represented in green.

In addition to sequence annotation at the phylum level for *Acidobacteria*, *Actinobacteria* and *Verrucomicrobia*, DNA sequences were classified into acidobacterial and verrucomicrobial classes as well as actinobacterial orders. A redundancy analysis of the relative abundance of these group-specific bacterial communities at the phylum level and deeper taxonomical resolutions showed that several *Acidobacteria* subgroups (4, 11, 17 and 21) were related to the chemical factors of the N+V-amended soils covered with a straw blanket (Fig 4). *Acidobacteria* subgroup 7 was related to the chemical factors of uncovered N-amended soils. Furthermore, *Actinobacteria* orders (*Rubrobacterales*, *Bifidobacteriales* and *Actinomycetales*) were related to the chemical factors of N+V-amended soils. *Opitutae*, a verrucomicrobial class, was related to the chemical factors of uncovered soils after three fertilizer applications with N and V (Fig 4).

## Discussion

This short-term study showed that the use of synthetic N and V as amendments may affect the MB and relative abundance of taxonomic groups of bacteria in sugarcane-cultivated soils through alterations of the soil chemical factors. Our results also revealed that sugarcane straw retention effects can be better described by taxonomic groups of bacteria than by MB.

Although long-term studies are commonly used to assess the effects of organic and inorganic fertilization and crop residue retention on the soil chemical and microbiological properties [4, 5, 30], short-term experiments are also important for understanding these effects, particularly on soil microbiota [31, 32]. However, there is a lack of information on the short-term impacts of fertilizer management practices on the microbiological properties of sugarcane-cultivated soils in Brazil.

Field studies have demonstrated that different sugarcane management strategies can alter the MB in soil [33, 34] as well as the soil bacterial community composition [35]. Our greenhouse short-term experiment showed that repeated N and V applications to the soil gradually inhibited soil MB. This effect can be related to soil chemical factors that are directly linked to the chemical composition of V, which is characterized by an acidic pH and high contents of organic C, K, calcium (Ca), magnesium (Mg) and sulfur [8].

Microbial biomass is the living component of soil organic matter, and it has long been suggested as a useful and sensitive measure of changes in the soil organic matter status [36, 37]. MB generally has been used to provide an early indication of changes in the organic matter content of a soil as a result of long-term variations in soil management [38–40]. Studies have also shown that soil MB is a more sensitive indicator of changing soil conditions than direct analysis of the total soil C content [33, 41]. Although the soil MB-C only constitutes 1–3% of the total soil C and the MB-N only constitutes up to 5% of the total soil N, they are the most labile C and N pools in soils [42]. Mooshammer et al. [43] provided evidence that C:N imbalances between resources and MB is compensated for by microbial C-use efficiency as well as by adaptations in microbial N-use efficiency. Thus, the more pronounced short-term changes observed in the soil MB compared with that of the total in soil C and N contents in our sugarcane-cultivated soils were consistent with these results from previous studies.

Vinasse is a source of nutrients, organic matter, and water, and its use can increase the productivity of sugarcane [44] with effects on the chemical [45, 46], physical [47], and biological [33, 48, 49] soil factors as well as greenhouse gas emissions [50]. Taken together, our results for soil MB, gas emissions and soil chemical factors in N- and N+V-amended soils provided additional evidence for previously reported results, which suggested that the highest content of MB was observed in soil treatments containing V as fertilizer [48]; highest CO_2_-C and N_2_O-N emissions were observed in sugarcane-cultivated soils with V and crop residue accumulation compared with N-amended soils [50]; increases in soil pH occurred as a short-term effect of periodical V application to the soil, and may to be linked to decreases in potential redox [46]; and increases in nutrient availability were also observed after V application to the soil [45].

The decrease in MB-C and MB-N was correlated to an increase in potassium and sulfur content as well as CO_2_-C and N_2_O-N emissions in the N+V-amended soils in the present study. The K concentration in the soil is a parameter used to calculate the V dosage to be applied in agricultural fields according to Brazilian legislation [51] because this residue has a high concentration of K. Concerning gas emissions from the soil, Jackson et al. [52] also showed a correlation between changes in MB-C and MB-N and CO_2_ and N_2_O emissions, respectively, from agricultural soil. Thus, our hypothesis that the changes in MB could be correlated with fertilizer-induced CO_2_-C and N_2_O-N emissions as well as chemical factors in sugarcane-cultivated soils is only supported for N+V-amendments and not for straw retention. Robertson and Thorburn [31] also showed that significant effects were not observed for management with sugarcane harvest residues on soil MB based on 1–2 year experiments. The effects of sugarcane crop residue on soil MB are expected to be more pronounced over longer time periods [53].

However, the responses of taxonomic groups in the soil bacterial community revealed fertilizer-induced and straw blanket-induced short-term effects in the sugarcane-cultivated soils. The increased availability of nutrients after repeated incorporation of N and V as fertilizer into the soil resulted in an increased abundance of *Actinobacteria* and decreased abundance of *Acidobacteria* and *Verrucomicrobia*. These findings correspond with results obtained in fields across N gradients [13], results from a long-term NPK fertilizer experiment [5], and results obtained with control conditions [54]. In these studies, a tradeoff in actinobacterial, acidobacterial and verrucomicrobial communities was explained by the dynamics of putative copiotrophic and oligotrophic bacteria.

Members of the phylum *Actinobacteria* are considered to have developed adaptations to nutrient-rich soils [13, 54, 55]. However, certain families belonging to *Actinobacteria* are also known to prefer soil environments with reduced C and nutrient availability [56, 57]; thus, they are considered putative oligotrophic. In turn, members of the phyla *Acidobacteria* and *Verrucomicrobia* have shown adaptations to low substrate concentrations in soil [58–60]. However, *Acidobacteria* subgroups 6 and 7 showed the opposite behavior in Amazon forest soils converted into agricultural fields, with their abundances linked to high contents of soil Ca, Mg, manganese (Mn) and boron (B) in soil [2]. In the present study, the increased abundance of *Actinobacteria* at the phylum level in N+V-amended soils, which resulted in the increased availability of nutrients because of the repeated incorporation of N and V as fertilizer, are consistent with a copiotrophic lifestyle, whereas decreases in the abundance of *Acidobacteria* and *Verrucomicrobia* in these soils are consistent with an oligotrophic lifestyle.

Orders belonging to *Actinobacteria* (*Rubrobacterales*, *Bifidobacteriales* and *Actinomycetales*), *Acidobacteria* subgroups (4, 11, 18, 22), and *Opitutae* class belonging to *Verrucomicrobia* were related to the chemical factors of soils fertilized with N+V compared with soils fertilized with only N and soils excluding any N and V fertilizer. The chemical factors of N+V-amended soils differed from the other treatments because of the high contents of sulfur, K and total C and values of pH. The N addition as fertilizer may decrease the decomposition of recalcitrant C [61], which may affect members of the phylum *Actinobacteria* because they are important decomposers and play a vital role in the C cycle [62]. Concerning the response of acidobacterial subgroups, subgroup 4 was also significantly higher in amended soils in the Amazon and had a positive effect on K content and exchangeable bases in the soil [2]. Bergmann et al. [63] showed that the generally oligotrophic phylum *Verrucomicrobia* benefits from C availability because of a slow-growing life strategy.

The straw blanket altered the soil bacterial community composition in N-amended, N+V-amended and control soils by increasing the abundance of *Acidobacteria* and *Verrucomicrobia* as well as by decreasing the abundance of *Actinobacteria*, which is an opposite pattern to what was revealed for soils without a straw blanket on the surface. Currently, little is known of the effects of sugarcane straw retention on soil microbial communities. However, previous studies based on genomic and culture traits indicated the use of carbon sources for *Acidobacteria* that span simple sugars to more complex substrates such as hemicellulose, cellulose, and chitin [60], and provided insights into their roles in organic carbon utilization in soil [64]. Isanapong et al. [65] showed genes coding for lignocellulosic degradation based on genomic analysis of an isolated member of *Verrucomicrobia*. In turn, decreased abundance of *Actinobacteria* in soil has been linked to decreased soil pH [66, 67]. This can explain decreased abundance of *Actinobacteria* as an effect of straw retention in our soils, which characterized lower soil pH. Although increase in abundance of γ-*Proteobacteria* in soils with a straw blanket was not confirmed by all of the molecular approaches used here to assess taxonomic groups of bacteria, previous results showed putative genes for production of organic acids involved with mineral solubilization based on genomic analysis of an isolated member of the γ-*Proteobacteria* [68]. However, a long-term experiment focused on the mineralization of crop residues added to soil is necessary to better understand its effects on soil bacterial community compositions.

Improvements in the organic matter content of soil and total C and N nutrition result from long-term straw accumulation, mainly in the surface soil [10, 69], which may explain why improvements were not observed in the organic matter, total C and total N when straw cover was used in the present study. However, straw addition is known to enhance drought resistance [70]. Buckley and Schmidt [55] reported that *Verrucomicrobia* is a bacterial phylum that is positively linked to soil moisture content. Hence, advantages in conserving soil moisture provided by the straw blanket may explain the high abundance of *Verrucomicrobia* found in soils with sugarcane straw at the surface.

Taken together, our results concerning bacterial community composition support the hypothesis that taxonomic groups of bacteria respond to fertilizer-induced and straw-blanket effects in sugarcane-cultivated soils.

In conclusion, our results obtained from a short-term greenhouse experiment provide evidence that MB is a relevant parameter in studies of the potential effects of V in combination with N fertilizer on microbiological properties of sugarcane-cultivated soils. In addition, our findings revealed that *Acidobacteria*, *Actinobacteria* and *Verrucomicrobia* are potential early-warning microbial bioindicators of the effects of N and V use as fertilizer on soil bacterial communities in sugarcane-cultivated soils, with *Actinobacteria* the best potential microbial bioindicator of straw-retention effects in these agricultural soils.

## Supporting Information

S1 File Python script used for heat map construction. (DOCX)

S1 Table File size in megabytes and number of sequencing reads obtained for each treatment over time in the greenhouse experiment (DOCX)

S2 Table Microbial biomass carbon and nitrogen, total carbon and nitrogen and organic matter (OM) determined in the topsoil layer (0–10 cm) of sugarcane-cultivated soils before fertilizing and on the maximum and minimum CO_2_-C and N_2_O-N emissions from soil over time in each of three applications of fertilizer (DOCX)

S3 Table Soil pH, sulfur, potassium and exchangeable bases determined in the topsoil layer (0–10 cm) at different experimental treatments before fertilizing and on the maximum and minimum CO_2_-C and N_2_O-N emissions from soil over time in each of three applications of fertilizer (DOCX)

S4 Table Relative abundance of bacterial phyla in sugarcane soils before fertilizing and on the maximum CO_2_-C and N_2_O-N emissions from soil over time in each of three applications of fertilizer (DOCX)
