# Supplementary material for: Multi-Analytical Approach Reveals Potential Microbial Indicators in Soil for Sugarcane Model Systems
Source: PLoS One. 2015 Jun 9;10(6):e0129765. doi: 10.1371/journal.pone.0129765 (PMC4461295; doi:10.1371/journal.pone.0129765)
Supplement: S1 File — (DOCX) [file pone.0129765.s001.docx]

**S1 File.** Python script used for heat map construction

#Taxonomic heat map based on the Euclidean distance

#Created by Genivaldo G.Z. Silva

#Input: FOCUS relative abundance of different samples

#Output: Heat map comparing different samples

def main(FOCUS_prediction):

F=FOCUS_prediction

import numpy as np

import numpy.linalg as np

import matplotlib

from pylab import *

f=open(F)

head=f.readline().replace("\n","").split("\t")[1:]

result=[]

for line in f:

if "Class___" in line:

result.append([float(x) for x in line.split("\t")[1:]])

f.close()

result=np.array(result)

result=result.transpose()

c=0

o=open("heat_"+F,"w+")

o.write("-\t"+"\t".join(head)+"\n")

for a in result:

c2=0

l=[]

for b in result:

dist = np.linalg.norm(a-b)

l.append(str(dist))

c2+=1

o.write(head[c]+"\t"+"\t".join(l)+"\n")

c+=1

o.close()

###################################################################

fig, ax = plt.subplots()

inputFile=open("heat_"+F)

row_labels=inputFile.readline().replace("\n","").split("\t")[1:]

data=[];yy=0;column_labels=[]

#read and store the data

for i in inputFile:

i=i.split("\t")

data.append(np.array([float(x) for x in i[1:]]))

xx=len(i)-1

column_labels.append(i[0])

yy+=1

inputFile.close()

data=np.array(data)

#scales

#http://wiki.scipy.org/Cookbook/Matplotlib/Show_colormaps

pcolor(data,cmap=matplotlib.cm.Blues,alpha=0.8,edgecolors='black')

colorbar()

axis([0,xx,0,yy])

# put the major ticks at the middle of each cell

ax.set_xticks(np.arange(data.shape[1])+0.3)

ax.set_yticks(np.arange(data.shape[0])+0.5)

#write the labels for the rows and columns

ax.set_xticklabels(row_labels,fontsize=2,rotation=90)

ax.set_yticklabels(column_labels,fontsize=2)

#Re-size the figure in order to fit the labels

DefaultSize = fig.get_size_inches()

fig.set_size_inches( (DefaultSize[0]/2, DefaultSize[1]/2))

# Turn off all the ticks

tick_params(length = 0)

#write the heat-map

savefig("heat_"+F+'.png',dpi=600)#)

close()

print "Done :)"

print "Please check "+"heat_"+F+'.png'

#main("result_merged.txt.xls")
