# Supplementary material for: Multi-Analytical Approach Reveals Potential Microbial Indicators in Soil for Sugarcane Model Systems
Source: PLoS One. 2015 Jun 9;10(6):e0129765. doi: 10.1371/journal.pone.0129765 (PMC4461295; doi:10.1371/journal.pone.0129765)
Supplement: S1 Table — (DOCX) [file pone.0129765.s002.docx]

**S1 Table.** File size in megabytes and number of sequencing reads obtained for each treatment over time in the greenhouse experiment

| Fertilizing | Treatments | Size megabytes^*^ | N. of merged sequence reads^*‡^ | N. of not merged sequence reads^*^ |
| --- | --- | --- | --- | --- |
| Before fertilizer amendment | | | | |
|  |  | 51.98 ± 8.68 | 77,278 ± 12,057 | 65,147 ± 10,801 |
|  |  |  |  |  |
| First fertilizer amendment (0 DAP) | | | | |
|  | N | 105.21 ± 33.43 | 170,571 ± 36,449 | 115,515 ± 40,043 |
|  | N+S | 146.58 ± 77.49 | 307,954 ±145,974 | 137,797 ± 78,930 |
|  | N+V | 129.67 ± 39.40 | 285,914 ± 98,865 | 119,885 ± 33,686 |
|  | N+V+S | 145.81 ± 78.09 | 149,788 ±115,593 | 148,739 ± 79,535 |
|  | C | 93.07 ± 26.39 | 90,930 ± 56,480 | 140,029 ± 55,345 |
|  | C+S | 122.12 ± 50.39 | 212,817 ±116,025 | 130,531 ± 39,513 |
|  |  |  |  |  |
| Second fertilizer amendment (150 DAP) | | | | |
|  | N | 143.83 ± 52.41 | 161,646 ± 45,267 | 191,277 ± 74,958 |
|  | N+S | 103.24 ± 19.73 | 145,699 ± 51,650 | 123,819 ± 33,647 |
|  | N+V | 115.95 ± 13.70 | 315,227 ± 24,330 | 99,072 ± 14,172 |
|  | N+V+S | 112.50 ± 66.47 | 243,234 ± 21,739 | 112,128 ± 49,951 |
|  | C | 77.94 ± 55.83 | 83,454 ± 47,730 | 94,744 ± 68,511 |
|  | C+S | 56.89 ± 28.75 | 77,271 ± 20,182 | 71,516 ± 44,002 |
|  |  |  |  |  |
| Third fertilizer amendment (210 DAP) | | | | |
|  | N | 131.68±28.87 | 268,238 ± 87,468 | 119,388 ± 15,553 |
|  | N+S | 136.25±45.29 | 280,168 ±142,636 | 121,574 ± 25,975 |
|  | N+V | 55.16±14.78 | 76,737 ± 7,995 | 65,121 ± 23,398 |
|  | N+V+S | 110.95±31.71 | 216,580 ± 78,245 | 101,667± 26,214 |
|  | C | 108.56±36.09 | 140,470 ± 88,178 | 112,154 ± 32,263 |
|  | C+S | 60.47±8.97 | 71,796 ± 20,310 | 78,546 ± 8,660 |

DAP = days after planting

* Post quality control on SeqClean script

‡ After merge paired reads using FLASH
