# Supplementary material for: Multi-Analytical Approach Reveals Potential Microbial Indicators in Soil for Sugarcane Model Systems
Source: PLoS One. 2015 Jun 9;10(6):e0129765. doi: 10.1371/journal.pone.0129765 (PMC4461295; doi:10.1371/journal.pone.0129765)
Supplement: S2 Table — (DOCX) [file pone.0129765.s003.docx]

**S2 Table.** Microbial biomass carbon and nitrogen, total carbon and nitrogen and organic matter (OM) determined in the topsoil layer (0-10 cm) of sugarcane-cultivated soils before fertilizing and on the maximum and minimum CO_2_-C and N_2_O-N emissions from soil over time in each of three applications of fertilizer

| Treatments | Before fertilizer  amendment  (0 DAP) |  | First fertilizer amendment | | |  | Second fertilizer amendment | | |  | Third fertilizer amendment | | |
| --- | --- | --- | --- | --- | --- | --- | --- | --- | --- | --- | --- | --- | --- |
|  |  |  | Maximum  gas emissions  (7 DAP) |  | Minimum  gas emissions  (150 DAP) |  | Maximum  gas emissions  (157 DAP) |  | Minimum  gas emissions  (210 DAP) |  | Maximum  gas emissions  (217 DAP) |  | Minimum  gas emissions  (250 DAP) |
|  |  | | | | | | | | | | | | |
|  | ------------------------------------------------------------------------ mg kg^-1^ Carbon-Microbial Biomass (dry soil) --------------------------------------------------------------------------- | | | | | | | | | | | | |
| N |  |  | 368.3*a^†^±49.9^‡^ |  | 269.6b±30,3 |  | 271.7a±45.6 |  | 195.8b±27.9 |  | 117.0a±11.5 |  | 103.3a±11.9 |
| N+S |  |  | 395.7a±18.7 |  | 317.4b±37,3 |  | 212.9a±59.6 |  | 196.8b±15.0 |  | 131.2a±17.8 |  | 120.6a±15.8 |
| N+V |  |  | 123.4b±24.2 |  | 448.4a±56,3 |  | 118.5b±28.3 |  | 288.5a±26.3 |  | 111.1b±26.7 |  | 198.5a±22.3 |
| N+V+S |  |  | 164.9b±23.0 |  | 395.4a±26,7 |  | 119.6b±23.1 |  | 201.2a±123.7 |  | 108.1b±28.2 |  | 200.4a±23.1 |
| C | 342.1±35.0 |  | 305.3a±24.3 |  | 275.5a±33,8 |  | 236.0a±24.9 |  | 188.0a±25.5 |  | 131.6a±16.9 |  | 98.7a±11.7 |
| C+S |  |  | 322.3a±14.4 |  | 267.3a±32,2 |  | 248.1a±24.9 |  | 168.6a±26.3 |  | 123.0a±17.5 |  | 110.9a±14.9 |
|  | ------------------------------------------------------------------------ mg kg^-1^ Nitrogen-Microbial Biomass (dry soil) ------------------------------------------------------------------------- | | | | | | | | | | | | |
| N |  |  | 44.8a±21.3 |  | 26.0b±9.1 |  | 54.9a±18.0 |  | 20.7b±7.5 |  | 24.4a±12.4 |  | 19.6a±4.9 |
| N+S |  |  | 43.2a±26.1 |  | 26.0b±3.9 |  | 52.4a±4.8 |  | 23.3b±2.1 |  | 34.8a±16.2 |  | 25.5a±5.5 |
| N+V |  |  | 105.4a±58.3 |  | 26.1b±12.1 |  | 99.9a±15.9 |  | 25.4b±3.0 |  | 55.5a±9.4 |  | 30.8b±5.2 |
| N+V+S |  |  | 109.1a±79.0 |  | 36.3b±4.8 |  | 95.1a±2.8 |  | 32.5b±2.7 |  | 61.0a±12.0 |  | 32.7b±4.9 |
| C | 62.3±1.8 |  | 58.9a±17.3 |  | 22.2b±4.8 |  | 21.4a±16.2 |  | 20.3a±6.0 |  | 20.1a±17.5 |  | 19.7a±5.6 |
| C+S |  |  | 58.9a±22.9 |  | 30.4b±16.6 |  | 28.2a±16.0 |  | 26.8a±11.5 |  | 25.2a±19.2 |  | 24.4a±8.1 |
|  | ------------------------------------------------------------------------------------- % total soil Carbon --------------------------------------------------------------------------------------------- | | | | | | | | | | | | |
| N |  |  | 2.19b±0.01 |  | 2.28a±0.02 |  | 2.28a±0.02 |  | 2.29a±0.05 |  | 2.29a±0.03 |  | 2.28a±0.04 |
| N+S |  |  | 2.19b±0.01 |  | 2.35a±0.01 |  | 2.30a±0.01 |  | 2.25b±0.02 |  | 2.21a±0.06 |  | 2.20a±0.05 |
| N+V |  |  | 2.19b±0.02 |  | 2.46a±0.01 |  | 2.48a±0.02 |  | 2.49a±0.01 |  | 2.50a±0.16 |  | 2.52a±0.02 |
| N+V+S |  |  | 2.13b±0.02 |  | 2.38a±0.01 |  | 2.40a±0.01 |  | 2.43a±0.02 |  | 2.48a±0.08 |  | 2.50a±0.06 |
| C | 2.2±0.2 |  | 2.04b±0.01 |  | 2.27a±0.01 |  | 2.26a±0.01 |  | 2.25a±0.03 |  | 2.24a±0.04 |  | 2.23a±0.01 |
| C+S |  |  | 2.07b±0.01 |  | 2.29a±0.04 |  | 2.27a±0.02 |  | 2.31a±0.04 |  | 2.33a±0.04 |  | 2.32a±0.02 |
|  | ------------------------------------------------------------------------------------- % total soil Nitrogen ------------------------------------------------------------------------------------------- | | | | | | | | | | | | |
| N |  |  | 0.21a±0.03 |  | 0.18a±0.02 |  | 0.20a±0.02 |  | 0.18a±0.02 |  | 0.19a±0.05 |  | 0.18a±0.02 |
| N+S |  |  | 0.20a±0.03 |  | 0.18a±0.01 |  | 0.19a±0.01 |  | 0.18a±0.01 |  | 0.17a±0.01 |  | 0.18a±0.01 |
| N+V |  |  | 0.23a±0.02 |  | 0.18a±0.02 |  | 0.24a±0.02 |  | 0.18a±0.02 |  | 0.25a±0.01 |  | 0.18a±0.02 |
| N+V+S |  |  | 0.22a±0.02 |  | 0.19a±0.03 |  | 0.23a±0.02 |  | 0.18a±0.02 |  | 0.23a±0.01 |  | 0.19a±0.03 |
| C | 0.18±0.02 |  | 0.17a±0.02 |  | 0.16a±0.02 |  | 0.15a±0.02 |  | 0.14a±0.01 |  | 0.14a±0.04 |  | 0.13a±0.02 |
| C+S |  |  | 0.17a±0.02 |  | 0.16a±0.01 |  | 0.15a±0.02 |  | 0.14a±0.01 |  | 0.14a±0.05 |  | 0.13a±0.01 |
|  | ------------------------------------------------------------------------------------- g.dm^-3^ Organic Matter ------------------------------------------------------------------------------------------ | | | | | | | | | | | | |
| N |  |  | 36.0a±1.0 |  | 28.1b±0.5 |  | 32.0a±0,81 |  | 27.2b±2.3 |  | 32.4a±2.0 |  | 28.1b±1.6 |
| N+S |  |  | 36.9a±2.5 |  | 28.9b±0.8 |  | 33.0a±1,41 |  | 28.1b±1.7 |  | 33.0a±1.5 |  | 29.0b±1.3 |
| N+V |  |  | 35.3a±1.6 |  | 27.7b±0.9 |  | 36.5a±0,57 |  | 27.1b±2.0 |  | 38.8a±3.6 |  | 28.7b±2.1 |
| N+V+S |  |  | 34.1a±1.5 |  | 28.2b±1.5 |  | 36.5a±2,08 |  | 27.3b±1.2 |  | 38.3a±1.7 |  | 27.3b±2.2 |
| C | 28.7±0.3 |  | 27.3a±1.7 |  | 26.0a±1.5 |  | 25.2a±1,25 |  | 25.1a±1.5 |  | 24.3a±1.3 |  | 23.7a±1.5 |
| C+S |  |  | 27.8a±1.9 |  | 26.7a±0.8 |  | 26.1a±1,73 |  | 25.3a±1.8 |  | 25.1a±1.7 |  | 24.8a±2.0 |

DAP = days after planting

N = nitrogen as fertilizer; V = *vinasse* as fertilizer; S = straw blanket; C = control - without any N and V fertilizer

*Average for each of three replicates of soil

Tukey’s test was performed separately for each experimental treatment and applications of fertilizer

†Values with the same letters were not significantly different (*p*<0.05) based on upon a Tukey’s test

‡Standard deviation of the average for each of three replicates of soil
