# Supplementary material for: Multi-Analytical Approach Reveals Potential Microbial Indicators in Soil for Sugarcane Model Systems
Source: PLoS One. 2015 Jun 9;10(6):e0129765. doi: 10.1371/journal.pone.0129765 (PMC4461295; doi:10.1371/journal.pone.0129765)
Supplement: S3 Table — (DOCX) [file pone.0129765.s004.docx]

**S3 Table.** Soil pH, sulfur, potassium and exchangeable bases determined in the topsoil layer (0-10 cm) at different experimental treatments before fertilizing and on the maximum and minimum CO_2_-C and N_2_O-N emissions from soil over time in each of three applications of fertilizer

| Treatments | Before fertilizer  amendment  (0 DAP) |  | First fertilizer amendment | | | |  | Second fertilizer amendment | | |  | Third fertilizer amendment | | |
| --- | --- | --- | --- | --- | --- | --- | --- | --- | --- | --- | --- | --- | --- | --- |
|  |  |  | Maximum  gas emissions  (7 DAP) |  | | Minimum  gas emissions  (150 DAP) |  | Maximum  gas emissions  (157 DAP) |  | Minimum  gas emissions  (210 days) |  | Maximum  gas emissions  (217 DAP) |  | Minimum  gas emissions  (250 DAP) |
|  |  | | | | | | | | | | | | | |
|  | ----------------------------------------------------------------------------------------pH ------------------------------------------------------------------------------------------------- | | | | | | | | | | | | | |
| N |  |  | 5.2*a^†^±0.1^‡^ |  | 5.2a±0.05 | |  | 5.2a±0.05 |  | 5.2a±0.1 |  | 5.0a±0.1 |  | 5.1a±0.1 |
| N+S |  |  | 5.1a±0.1 |  | 5.2a±0.05 | |  | 5.1a±0.1 |  | 5.2a±0.1 |  | 5.1a±0.1 |  | 5.2a±0.1 |
| N+V |  |  | 5.6b±0.1 |  | 5.9a±0.1 | |  | 5.5b±0.5 |  | 5.8a±0.05 |  | 5.6b±0.1 |  | 5.8a±0.1 |
| N+V+S |  |  | 5.4b±0.1 |  | 5.6a±0.1 | |  | 5.4b±0.1 |  | 5.7a±0.1 |  | 5.5b±0.1 |  | 5.7a±0.1 |
| C | 5.4±0.2 |  | 5.3a±0.1 |  | 5.4a±0.1 | |  | 5.3a±0.1 |  | 5.4a±0.1 |  | 5.4a±0.1 |  | 5.5a±0.1 |
| C+S |  |  | 5.2a±0.1 |  | 5.3a±0.1 | |  | 5.2a±0.1 |  | 5.3a±0.1 |  | 5.3a±0.1 |  | 5.4a±0.1 |
|  | ------------------------------------------------------------------------------Sulfur (mg.dm^-3^) -------------------------------------------------------------------------------- | | | | | | | | | | | | | |
| N |  |  | 7.1a±2.2 |  | 9.5a±3.3 | |  | 9.0a±2.7 |  | 9.5a±2.7 |  | 23.7a±7.7 |  | 24.7a±9.9 |
| N+S |  |  | 6.3a±1.9 |  | 8.0a±3.1 | |  | 8.5a±2.2 |  | 8.9a±2.3 |  | 14.0a±4.2 |  | 15.6a±6.5 |
| N+V |  |  | 49.0b±6.3 |  | 74.1a±5.5 | |  | 185.4b±7.5 |  | 215.3a±8.6 |  | 270.2b±9.2 |  | 302.4a±9.9 |
| N+V+S |  |  | 22.1b±7.0 |  | 38.4a±7.1 | |  | 97.3b±6.5 |  | 113.4a±7.8 |  | 174.2b±8.4 |  | 207.6a±6.8 |
| C | 6.1±0.2 |  | 6.2a±1.1 |  | 6.6a±1.6 | |  | 7.1a±1.7 |  | 7.8a±1.9 |  | 21.7a±3.2 |  | 22.3a±3.7 |
| C+S |  |  | 5.0a±1.0 |  | 5.0a±2.1 | |  | 6.0a±1.4 |  | 6.8a±1.7 |  | 19.5a±3.1 |  | 20.1a±3.3 |
|  | ------------------------------------------------------------------------------ Potassium (mmolc dm^-3^)--------------------------------------------------------------------------------- | | | | | | | | | | | | | |
| N |  |  | 2.1±0.1 |  | 2.9±0.8 | |  | 3.6±0.6 |  | 4.3±1.2 |  | 5.5±1.6 |  | 5.7±1.2 |
| N+S |  |  | 2.1±0.1 |  | 2.2±0.8 | |  | 3.7±0.7 |  | 4.2±1.2 |  | 5.1±1.0 |  | 5.5±1.1 |
| N+V |  |  | 11.3a±0.8 |  | 13.0a±3.5 | |  | 15.6a±3.1 |  | 18.3a±3.3 |  | 40.8a±10.0 |  | 42.3a±11.1 |
| N+V+S |  |  | 4.9a±0.9 |  | 5.8a±1.2 | |  | 11.3a±1.7 |  | 12.5a±1.8 |  | 32.3a±3.9 |  | 33.7a±4.2 |
| C | 2.1±0.2 |  | 2.2a±0.1 |  | 2.5a±0.3 | |  | 3.8a±0.4 |  | 4.1a±1.1 |  | 4.5a±1.8 |  | 4.6a±1.3 |
| C+S |  |  | 2.4a±0.2 |  | 2.5a±0.4 | |  | 3.0a±0.6 |  | 3.1a±0.9 |  | 3.4a±1.8 |  | 4.0a±1.4 |
|  | -----------------------------------------------------------------------------------EB (mmolc kg^-1^)-------------------------------------------------------------------------------------- | | | | | | | | | | | | | |
| N |  |  | 71.8a±3.3 |  | 76.2a±5.5 | |  | 81.1a±4.3 |  | 85.6a±5.1 |  | 92.8a±3.5 |  | 94.3a±3.0 |
| N+S |  |  | 74.7a±1.8 |  | 70.8a±3.4 | |  | 78.8a±3.6 |  | 84.7a±6.7 |  | 91.1a±3.6 |  | 95.7a±4.5 |
| N+V |  |  | 72.2a±2.3 |  | 71.5a±2.1 | |  | 79.9a±3.0 |  | 85.0a±5.5 |  | 85.6a±9.1 |  | 89.4a±7.8 |
| N+V+S |  |  | 69.6a±2.3 |  | 66.4a±1.7 | |  | 72.2a±4.1 |  | 81.5a±6.1 |  | 89.0a±5.4 |  | 91.2a±6.3 |
| C | 77.9±2.0 |  | 78.4a±4.3 |  | 84.3a±11.0 | |  | 98.9a±10.7 |  | 112.7a±11.7 |  | 130.6a±7.1 |  | 134.6a±7.5 |
| C+S |  |  | 71.4a±2.6 |  | 75.5a±3.9 | |  | 92.3a±9.5 |  | 108.5a±10.0 |  | 129.6a±5.2 |  | 132.1a±7.0 |

DAP = days after planting

N = nitrogen as fertilizer; V = *vinasse* as fertilizer; S = straw blanket; C = control - without any N and V fertilizer; EB = exchangeable bases - the sum of Ca, Mg and K

*Average for each of three replicates of soil

Tukey’s test was performed separately for each experimental treatment and applications of fertilizer

†Values with the same letters were not significantly different (*p*<0.05) based on upon a Tukey’s test

‡Standard deviation of the average for each of three replicates of soil
