# Supplementary material for: Multi-Analytical Approach Reveals Potential Microbial Indicators in Soil for Sugarcane Model Systems
Source: PLoS One. 2015 Jun 9;10(6):e0129765. doi: 10.1371/journal.pone.0129765 (PMC4461295; doi:10.1371/journal.pone.0129765)
Supplement: S4 Table — (DOCX) [file pone.0129765.s005.docx]

**S4 Table**. Relative abundance of bacterial phyla in sugarcane soils before fertilizing and on the maximum CO_2_-C and N_2_O-N emissions from soil over time in each of three applications of fertilizer

| **Bacterial groups** |  | **Before fertilizing** |  | **First fertilizer amendment (0 DAP)** | | | |  | **Control** | |
| --- | --- | --- | --- | --- | --- | --- | --- | --- | --- | --- |
|  |  |  |  | **N** | **N+S** | **N+V** | **N+V+S** |  | **C** | **C+S** |
| *Acidobacteria* |  | 2.8* ± 0.9^†^ |  | 2.2 a^‡^A^§^±0.2 | 3.1 aA ± 0.2 | 2.7 aA ± 0.2 | 2.4 aA ± 0.8 |  | 2.2 aA ± 1.9 | 2.8 aA ±0.2 |
| *Actinobacteria* |  | 32.6 ± 1.3 |  | 33.5 aA±0.3 | 31.4 aA±0.6 | 33.8 aA ±1.8 | 33.8 aA±2.3 |  | 31.6 aA±0.6 | 33.2aA±0.7 |
| *Bacteroidetes* |  | 2.9 ± 0.1 |  | 2.5 aB ± 0.2 | 2.3 aA±0.06 | 2.4aB ± 0.04 | 2.3 aA ± 0.2 |  | 3.5 aA ± 0.4 | 2.3 bA ±0.2 |
| *Chloroflexi* |  | 1.3 ± 0.3 |  | 1.1 aA ± 1.0 | 1.0 aA ± 0.1 | 0.9 aA ± 0.1 | 0.9 aA ± 0.2 |  | 0.8 aA ± 0.1 | 1.2 aA ±0.2 |
| *Cyanobacteria* |  | 0.5 ± 0.2 |  | 0.5 aA±0.04 | 0.5 aA ± 0.1 | 0.5aA ± 0.03 | 0.7 aA ± 0.3 |  | 0.7 aA ± 0.1 | 0.7 aA ±0.1 |
| *Deinococcus-Thermus* |  | 3.9 ± 1.1 |  | 4.2 aA ±0.1 | 3.8 aA ± 0.2 | 3.8aA ± 0.04 | 3.7 aA ± 0.4 |  | 4.2 aA±1.05 | 3.4 aA ±0.4 |
| *Firmicutes* |  | 3.4 ± 0.2 |  | 4.0 aA±0.04 | 4.7 aA ± 0.4 | 4.4 aA ± 0.4 | 4.4 aA ± 0.3 |  | 4.2 aA ± 0.5 | 4.2 aA ±0.5 |
| *Planctomycetes* |  | 12.0 ± 0.3 |  | 12.2 aA±0.2 | 12.2 aA±0.1 | 12.9 aA ±1.0 | 11.3 aA±1.5 |  | 12.6 aA±1.1 | 11.4aA±0.6 |
| α-*Proteobacteria* |  | 11.0 ± 0.6 |  | 10.2 aA±0.6 | 11.4 aA±0.4 | 10.5aA ± 0.8 | 11.1 aA±0.3 |  | 10.9 aA±1.8 | 11.3aA±0.5 |
| β-*Proteobacteria* |  | 10.5 ± 0.6 |  | 10.8 aA±0.2 | 10.2 aA±0.1 | 9.9 aA ± 0.2 | 11.1 aA±1.4 |  | 10.4 aA±0.7 | 10.4aA±0.6 |
| δ-*Proteobacteria* |  | 12.6 ± 0.7 |  | 13.1 aA±0.5 | 12.5 aA±0.4 | 11.4 aA ±0.3 | 13.2 aA±1.4 |  | 13.2 aA±1.4 | 12.9aA±0.2 |
| γ-*Proteobacteria* |  | 0.2 ± 0.01 |  | 0.2 aA ± 0.1 | 0.4 aA ± 0.1 | 0.6 aB ± 0.1 | 0.3 aA±0.02 |  | 0.1 aA±0.05 | 0.1aA±0.07 |
| *Spirochaetes* |  | 0.6 ± 0.1 |  | 0.3 aA ± 0.1 | 0.5 aA ± 0.1 | 0.6 aA ± 0.1 | 0.1aA ±0.03 |  | 0.4 aA ± 0.1 | 0.3 aA ±0.1 |
| *Tenericutes* |  | 0.8 ± 0.07 |  | 0.8 aA±0.03 | 0.9 aA ± 0.1 | 0.8 aA ± 0.1 | 0.6 aA ± 0.2 |  | 0.6 aA ± 0.2 | 0.7 aA ±0.2 |
| *Verrucomicrobia* |  | 3.6 ± 0.3 |  | 3.3 aA ± 0.2 | 3.6 aA ± 0.1 | 3.2 aA ± 0.2 | 3.3 aA ± 0.8 |  | 3.6 aA ± 0.1 | 4.0 aA ±0.3 |
| Others |  | 1.0 ± 0.4 |  | 0.7 aA±0.04 | 1.0 aA ± 0.2 | 1.1 aA ± 0.2 | 0.5 aA ± 0.2 |  | 0.6 aA ± 0.1 | 0.9 aA ±0.1 |
|  | | |  | **-------------Second fertilizer amendment (150 DAP) ---------** | | | |  | **-----------Control------------** | |
| *Acidobacteria* | | |  | 3.3 aA ±0.2 | 3.9 aB ± 0.3 | 1.7 aA ± 0.9 | 2.8 aB ± 0.7 |  | 2.8 aA±0.72 | 4.7 bA ± 0.5 |
| *Actinobacteria* | | |  | 29.1aA±1.1 | 26.6aA**±**0.6 | 37.6 aB± 3.5 | 32.4 aB±2.7 |  | 31.1aA±3.0 | 24.7 bA±1.2 |
| *Bacteroidetes* | | |  | 2.9 aA ±0.1 | 2.9 aA ±0.3 | 3.2 aA ± 0.4 | 3.2 aA ± 0.5 |  | 2.9 aA ± 0.4 | 3.2 aA ±0.5 |
| *Chloroflexi* | | |  | 0.8 aA ±0.1 | 0.9 aA ±0.5 | 0.1 aA ±0.01 | 0.8 aA ± 0.2 |  | 0.9 aA ± 0.4 | 0.6 aA ± 0.2 |
| *Cyanobacteria* | | |  | 0.5 aA ±0.1 | 0.4 aA ±0.1 | 0.1 aB± 0.01 | 0.2 aA ± 0.1 |  | 0.6 aA ± 0.2 | 0.4 aA ± 0.2 |
| *Deinococcus-Thermus* | | |  | 5.0 aA ±0.2 | 4.9 aA ±0.2 | 5.3 aA ± 0.1 | 5.0 aA ± 0.2 |  | 4.3 aA ± 1.3 | 4.6 aA ± 0.2 |
| *Firmicutes* | | |  | 4.4 aA ±0.2 | 4.8 aA ±0.5 | 4.1 aA ± 0.7 | 4.0 aA ± 0.3 |  | 4.3 aA ± 0.4 | 4.8 aA ± 1.0 |
| *Planctomycetes* | | |  | 13.7aA±0.1 | 13.8aA±0.5 | 12.6 aA ±0.7 | 12.5 aA±0.1 |  | 13.2aA± 2.0 | 14.0 aA±0.3 |
| α-*Proteobacteria* | | |  | 11.2aA±0.4 | 11.9aA±0.3 | 11.5 aA ±1.2 | 12.9 aA±0.9 |  | 10.8 aA±1.5 | 13.3 aA±0.8 |
| β-*Proteobacteria* | | |  | 11.2aA±0.1 | 11.1aA±0.4 | 11.4 aA ±0.4 | 12.2 aA±0.2 |  | 10.8 aA±0.1 | 11.6 aA±1.9 |
| δ-*Proteobacteria* | | |  | 11.9aA±0.3 | 11.9aA±0.7 | 8.4 aB ± 1.1 | 9.8 aA ±0.5 |  | 12.8 aA±1.2 | 11.2 aA±1.2 |
| γ-*Proteobacteria* | | |  | 0.9 aA ±0.2 | 1.0 aA ±0.1 | 0.9 aA ± 0.1 | 1.1 aA ±0.1 |  | 0.5 aA±0.31 | 1.2 bA ± 0.2 |
| *Spirochaetes* | | |  | 0.001 aA±0 | 0.07aA±0.0 | 0 aA ± 0 | 0.02 aA ± 0 |  | 0.1 aA ± 0 | 0.1 aA ± 0 |
| *Tenericutes* | | |  | 1.0aA±0.04 | 1.1 aA ±0.2 | 1.1 aA ± 0.2 | 1.0 aA±0.05 |  | 0.8aA ± 0.3 | 1.0 aA ± 0.1 |
| *Verrucomicrobia* | | |  | 3.0 aA ±0.4 | 3.3 aA ±0.3 | 0.4 aB ± 0.1 | 0.8 bB ± 0.1 |  | 3.4 aA ± 0.9 | 3.1 aA ± 0.4 |
| Others | | |  | 0.4 aA ±0.1 | 0.4 aA ±0.1 | 0.4 aA ± 0.2 | 0.4 aA ± 0.2 |  | 0.3aA± 0.01 | 0.6 aA ± 0.1 |
|  | | |  | **--------------Third fertilizer amendment (210 DAP)----------** | | | |  | **------------Control-----------** | |
| *Acidobacteria* | | |  | 1.2 aA ± 0.2 | 3.4 bA ± 0.5 | 1.2 aB ± 0.8 | 2.8 bB ± 0.6 |  | 2.6 aA ± 0.1 | 4.5 bA ± 0.8 |
| *Actinobacteria* | | |  | 35.5aA± 2.6 | 28.2 aB ± 0.4 | 38.5 aB ±5.6 | 31.0bB± 0.7 |  | 31.5aA ±0.7 | 25.9 bA±1.5 |
| *Bacteroidetes* | | |  | 1.6 aA ± 0.1 | 1.7 aA ± 0.1 | 2.2 aA ± 0.4 | 1.6 aA ± 0.3 |  | 2.0 aA ± 0.2 | 2.5 aA ±0.6 |
| *Chloroflexi* | | |  | 0.4 aA ± 0.1 | 0.4 aA ± 0.1 | 0.8 aA ± 0.3 | 0.5 aA ± 0.1 |  | 0.4 aA ± 0.1 | 0.5 aA ±0.1 |
| *Cyanobacteria* | | |  | 0.6 aB ± 0.1 | 0.7 aA ± 0.1 | 0.4 aB± 0.04 | 0.3 aB ± 0.1 |  | 1.0 aA ± 0.1 | 0.8 aA ±0.1 |
| *Deinococcus-Thermus* | | |  | 4.2 aA ± 0.1 | 3.4 aA ± 0.2 | 4.2 aA ± 0.2 | 3.9 aA ± 0.2 |  | 3.3 aA ± 0.5 | 3.2 aA ± 0.1 |
| *Firmicutes* | | |  | 4.4 aB ± 0.4 | 5.6 aA ± 0.2 | 3.4 aB ± 0.4 | 3.8 aB ± 0.1 |  | 5.1 aA ± 0.7 | 5.4 aA ± 0.4 |
| *Planctomycetes* | | |  | 12.6aA± 0.6 | 12.6 aA ± 0.4 | 8.8 aB ± 1.0 | 10.1aB± 0.8 |  | 12.6aA± 0.9 | 12.5 aA±0.6 |
| α-*Proteobacteria* | | |  | 10.2 aA±1.1 | 13.0 aA ± 0.4 | 13.1aA ± 2.2 | 16.3 bA±0.8 |  | 12.2 aA±0.4 | 14.1 aA±0.6 |
| β-*Proteobacteria* | | |  | 9.7 aA ± 0.4 | 10.9 aA ± 1.1 | 13.6 aB ±0.6 | 14.5 aB±0.5 |  | 9.6 aA ± 1.0 | 10.4aA±0.5 |
| δ-*Proteobacteria* | | |  | 13.9aA± 0.5 | 13.3 aA ± 0.2 | 9.6 aB ± 0.8 | 10.7 aB±0.3 |  | 13.6 aA±0.6 | 13.3aA±1.2 |
| γ-*Proteobacteria* | | |  | 0.1aA±0.05 | 0.4 bA ± 0.1 | 0.5 aA ± 0.1 | 0.9 bA ± 0.1 |  | 0.1 aA ± 0.1 | 0.3bA±0.04 |
| *Spirochaetes* | | |  | 0 aA ± 0 | 0aA ± 0 | 0 aA ± 0 | 0 aA ± 0 |  | 0 aA ± 0 | 0 aA ± 0 |
| *Tenericutes* | | |  | 1.1 aA ± 0.1 | 1.1 aA ± 0.1 | 0.8 aA ± 0.1 | 0.9 aA ± 0.1 |  | 0.9 aA ± 0.2 | 0.9 aA ± 0.1 |
| *Verrucomicrobia* | | |  | 3.5 aA ± 0.3 | 4.2 bA ± 0.2 | 0.8 aB ± 0.5 | 1.8 bB ± 0.4 |  | 4.4 aA ± 0.7 | 4.4 aA ± 0.1 |
| Others | | |  | 0.7 aA ± 0.1 | 0.8 aA ± 0.2 | 0.5 aA ± 0.1 | 0.4 aA ± 0.1 |  | 0.7 aA ± 0.2 | 0.8 aA ± 0.3 |

DAP = days after planting

N, nitrogen as fertilizer; V, *vinasse* as fertilizer; S, straw blanket; C, control - without any N and V fertilizer

*Average for each of three replicates of soil

†Standard deviation of the average for each of three replicates of soil

Tukey’s test was performed separately for each of three fertilizer applications. Samples with and without straw blanket were contrasted for treatments equally

fertilized (‡), and also for fertilized treatments and control soils under the same straw blanket condition (§)

Values with the same lower or upper-case letters were not significantly different (*p*<0.05) based on upon a Tukey’s test between contrasted samples
